# The Impact of the COVID-19 Pandemic Quarantine on Adults with Autism Spectrum Disorders and Intellectual Disability: A Longitudinal Study

**DOI:** 10.1007/s10803-022-05792-9

**Published:** 2022-10-30

**Authors:** Marina Jodra, Domingo García-Villamisar

**Affiliations:** Department of Personality, Evaluation and Clinical Psychology, Universidad Complutense de Madrid, Rector Royo Villanova s/n, Ciudad Universitaria, 28040 Madrid, Spain

**Keywords:** Autism Spectrum Disorders, COVID-19, Pandemy, Longitudinal study, Intellectual disability, Adults, Longitudinal data analysis, Environmental risk factors, Executive functioning, Motor (control, system)

## Abstract

The impact of the pandemic is being very significant psychologically, especially for people who were already vulnerable in these aspects, such as adults with Autism Spectrum Disorders (ASD) and Intellectual Disability (ID). A longitudinal analysis of motor aspects such as balance and gait, executive functions in daily life, severity of symptoms characteristic of autism, and degree of subjective well-being was performed in 53 adults with ASD and ID. A repeated measures ANOVA was performed and three measures were taken, the first in December 2019, the second in March 2020, and the last in July 2020. The results demonstrated a significant decrease in balance on the latter measure, along with a deterioration in well-being and ASD symptoms in the period of seclusion and an improvement in executive functions after seclusion.

## Introduction

The COVID 19 pandemic caused the Spanish government to decree a state of alarm on March 14, 2020, limiting the free movement of citizens and forcing home confinement. After forty-two days, on April 26, the "de-escalation plan" began, allowing a gradual return to the "new normality".

The impact of the COVID 19 Coronavirus pandemic is being very significant at health, and psychological levels. Some studies have detected a significant psychopathological impact on the general population (Chen et al., 2020; Duan & Zhu, 2020; Li et al., 2020; Yang et al., 2020) and population with previous psychiatric diagnoses where this impact was greatest (Colizzi et al., 2020; Kwong et al., 2021; Varma et al., 2021).

Regarding the impact of the pandemic on people with disabilities and chronic illnesses, a close link between stress and coping strategies such as the ability to distract oneself, denial, religion or blame has also been observed (Umucu & Lee, 2020). Individuals with Autism Spectrum Disorders (ASD), are among the population especially vulnerable to pandemic and anxiety (Baweja et al., 2022). Various research has observed higher levels of stress associated with the severity of symptoms characteristic of the disorder (i.e., Mayes et al., 2011; Hallett et al., 2013).

The population with ASD is more likely to have other comorbid disorders such as anxiety, depression (i.e., Hollocks et al., 2019; Vasa et al., 2020) or intellectual disability (Maenner et al., 2020), motor difficulties (Lim et al., 2021), premature aging (Roestorf et al., 2019), executive dysfunction (i.e., Demetriou et al., 2019; Geurts et al., 2014; Hill, 2004; Kaur & Pany, 2020) and adaptive behavior (Di Rezze et al., 2019). This makes adults with ASD and intellectual disabilities especially vulnerable to the effects of the pandemic and confinement on their quality of life. There are few studies on the specific effects of the pandemic on adults with ASD, although some results suggest that 72% of this population has experienced a deterioration in their mental health during this health emergency (Davidson et al., 2021). Difficulties in executive functions to plan, switch tasks, and inhibit the execution of stereotypical or obsessive routines make day-to-day living during confinement especially challenging for anyone with ASD (Baweja et al., 2022).

Confinement, closures of community settings and non-essential health services, and social distancing rules left parents, caregivers, and other natural supports trying to meet most of the needs of these individuals, leaving greatly reduced options (Eshraghi et al., 2020), many professionals struggled to offer their services remotely, and many needs were left to wait for the pandemic to end (Baweja et al., 2022).

The objective of this research is to inquire into the effects that confinement has had on this population in order to make adjustments to current interventions. As a consequence of the data analyzed, it is hypothesized that confinement and pandemic affect adults with ASD and ID, showing impaired balance and gait, increased executive dysfunction in daily life, impaired symptomatology and reduced subjective well-being. The research questions under analysis are:

1. Has confinement had an impact on participants' gait and balance?
2. Is there a worsening of the core symptomatology of ASD produced by the confinement?
3. Is there a deterioration of executive functions after a period of confinement
4. How has the pandemic affected the well-being of this population?

## Method

### Participants

The study has been conducted with a sample consisting of 53 subjects with ASD and ID (17 females and 36 males), ages 20–58 (*M* = 34.75). Table 1 shows data on the degree of intellectual disability, assessed through the Leiter International Performance Scale (LIPS; Leiter, 1948), and chronological age. As for the degree of intellectual disability, the IQ was taken into account, being 0 (light or slight) an IQ between 56–75, 1 (moderate) between 46–55, 2 (severe) between 26–45 and 3 (profound) between 0–25 (Schalock et al., 2021).

**Table 1:** Subject demographics and baseline characteristics

Sex		Male	Female	Total
	n	36	17	53
Chronological age	*M*	32.94	38.58	34.75
	*SD*	8.93	5.88	8.45
Grade of intellectual disability	*M*	1.83	2.11	1.92
	*SD*	1.08	.78	.99

Grade of intellectual disability; 0 = light or slight (IQ 56–75), 1 = moderate (IQ 46–55), 2 = serious (26–45), 3 = deep (0–25)

Participants were recruited from a day care programs for adults with special needs in Madrid and Vigo (Spain). Study participants remained in their confined residences, with a small group of peers. During this period, activities were limited to the maintenance of some day-to-day activities and short walks. From April 26, after confinement, they returned to their individual day center activity plan, with physical activities, physiotherapy, occupational therapy, communication, social skills, cognitive development, occupational workshops and leisure activities.

All participants were clinically diagnosed with autism by a psychiatrist or clinical psychologist with several years of experience in assessment of ASD and related conditions. Informed consent was provided by the participants or their guardians. Individuals from whom we could not obtain consent were excluded from the study (only one person was excluded for this reason). The ethics commission of the Nuevo Horizonte Association reviewed and approved this study.

The study was conducted during the pandemic, so the only exclusion criterion was that the person had been infected by COVID during data collection, or had spent periods of confinement outside the centers.

### Materials

#### Diagnostic Behavioral Assessment for Autism Spectrum Disorder- Revised (DiBAS-R; Sappok et al., 2014)

The DiBAS-R is a screening scale to assess autistic traits in adults with ID. It measures the two symptomatological domains of ASD established by the DSM-5 (APA, 2013); social communication and restrictive and repetitive patterns of behavior, interests or activities.

The instrument is composed of 19 items measured through a Likert-type scale, and rated at (3) always, (2) often, (1) sometimes, (0) never/no. Higher scores suggest greater severity of ASD traits (Sappok et al., 2014). The cut-off point is set at 20 points, so that scores above that score indicate the presence of possible ASD (Heinrich et al., 2018).

The DiBAS-R enjoys very good psychometric properties. The internal consistency of the total scale is high (α = 0.91), as is that of its two dimensions; 0.91 for the communication and interaction scale and 0.84 for the stereotypies, rigidity, and sensory disturbances scale (Heinrich et al., 2018; Sappok et al., 2014).

#### Dysexecutive Questionnaire (DEX; Burgess et al., 1996)

This questionnaire consists of 20 questions that aim to assess dysexecutive functions in everyday life. It consists of 5 factors (inhibition, executive memory, intentionality, positive affect and negative affect) (Burgess et al., 1998) and has good psychometric properties with a Cronbach's alpha of 0.91 (Wilson et al., 1996). In the adaptation of the questionnaire to the Spanish population (DEXSp) a Cronbach's alpha of 0.87 was obtained (Pedrero-Pérez et al., 2011). High scores on this questionnaire show the presence of greater executive difficulties.

The following cut-off points are proposed: < 10, optimal functioning; 10–18, functioning within normality; 19–28, moderately dysexecutive functioning that requires identification of possible causes, and > 28, significant dysexecutive impairment that would include serious pathologies (Bodenburg & Dosplaff, 2008).

#### The Tinetti Assessment Tool (TAT; Tinetti, 1986)

The Tinetti scale was developed to assess mobility and balance in the elderly and consists of two dimensions: balance and gait. For gait, the interviewer walks behind the patient and asks the patient to answer questions related to ambulation. To assess balance, the interviewer stands next to the patient, facing forward and to the right, without losing sight of the situation. The maximum score for gait is 12 points and for balance 16 points; the total sum of the scale is 28 points. High risk of falls is considered to be less than 19 points; risk of falls is 19 to 23 points; and low or mild risk is 24 to 28 points.

#### Personal Wellbeing Index (PWI); Cummins & Lau, 2005)

The Personal Wellbeing Index (PWI) of Cummins and Lau (2005) will be applied. The PWI is a brief 7-item questionnaire that is very useful and valid for the evaluation of emotional well-being in people with autism and disability. Each item is assessed on a scale from 0 to 10, where 0 is "very dissatisfied" and 10 is "very satisfied”. This questionnaire has demonstrated good reliability and validity (McGillivray et al., 2009).

#### Leiter International Performance Scale (LIPS; Leiter, 1948)

The non-verbal mental age of the participants was assessed using the Leiter test (Leiter, 1948), which measures cognitive functioning by means of tests that do not require the use of language, neither by the examiner nor by the person performing the test. This is composed of 54 subtests, divided into three blocks. The results provide the IQ, with 0 (mild) being an IQ between 56–75, 1 (moderate) between 46–55, 2 (severe) between 26–45 and 3 (profound) between 0–25 (Schalock et al., 2021). It shows satisfactory internal consistency with a reliability of 0.91 (Shah & Holmes,^ 1985^; Sharp,^ 1958^), and is very suitable for people with ID (Tsatsanis et al., 2003).

### Procedure

The variables relating to walking and balance were assessed in quiet rooms and taking into account the personal characteristics of each participant, allowing for the necessary breaks, and some of the tasks were explained using the communication systems used by each individual (pictograms, photographs, sign language, etc.)

The rest of the questionnaires were completed by the tutor of each person in each association, all of whom are professionals trained in psychological diagnosis and familiar with the study population, but not with the objectives of the research.

### Statistical Analysis

Data analysis was performed using the IBM SPSS Statistics 25 program for Windows.

A repeated measures ANOVA was performed to examine the impact of pandemic on severity of autism traits, executive dysfunction, balance, gait, and well-being. The Bonferroni test was used as a post-hoc test. Three measures were taken, the first in December 2019, the second in March 2020, and the last in July 2020.

## Results

Means and standard deviations (SD), at the three measurement times (T1, T2 and T3), for executive dysfunction (DEX), severity of autistic traits (DIBAS-R), emotional well-being (PWI), gait and balance (Tinetti) are shown in Table 2.

**Table 2:** Means and standard deviations (SD for executive dysfunction (DEX), severity of autistic traits (DIBAS-R), emotional well-being (PWI), gait and balance (Tinetti)

	T1		T2		T3	
	Mean	SD	Mean	SD	Mean	SD
DEX	47.31	11.79	47.16	10.84	43.00	9.94
DIBAS-R	18.45	6.78	21.27	7.69	19.11	5.73
TINETTI-BALANCE	10.06	4.03	10.18	3.68	8.93	4.51
TINETTI-WALKING	8.40	3.13	8.43	3.11	8.68	3.35
PWI	48.48	10.09	46.41	9.19	50.12	7.13

*DEX* Dysexecutive Questionnaire, *DIBAS-R* Diagnostic Behavioral Assessment for Autism Spectrum Disorder- Revised, *TINETI-BALANCE and TINETI-WALKING* The Tinetti Assessment Tool, *PWI* Personal Wellbeing Index

The results demonstrated a significant effect of time on the severity of ASD traits, F_(2,50)_ = 6.730, *p* < 0.010, $\eta$^2^ = 0.119; executive dysfunction, F_(2,47)_ = 8.534, *p* = 0.000, $\eta$^2^ = 0.154; balance, F_(2,43)_ = 4.513, *p* < 0.050, $\eta$^2^ = 0.095; and well-being, F_(2,38)_ = 4.443, *p* < 0.050, $\eta$^2^ = 0.105.

**Fig. 1:**
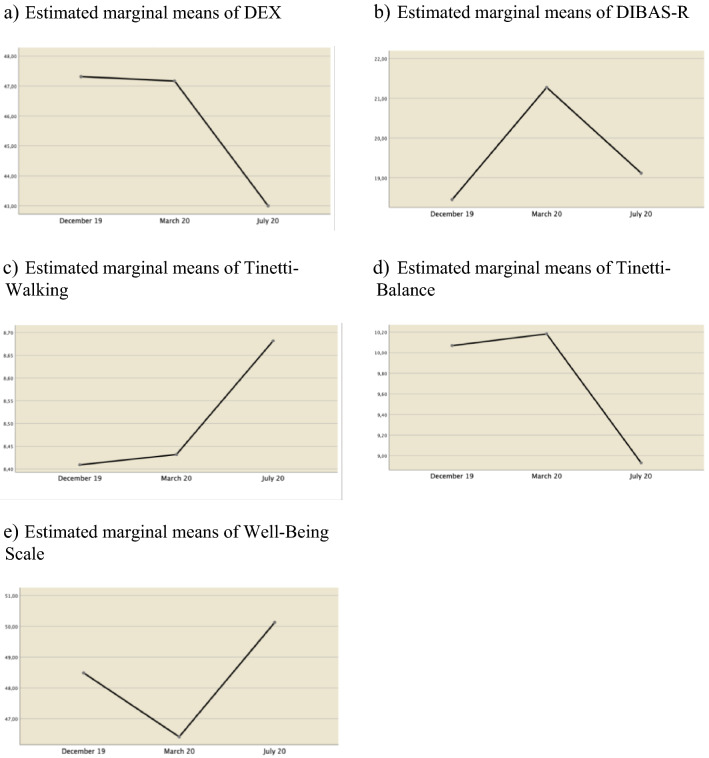
Executive dysfunction, autism traits severity, balance, gait and well-being development during COVID-19 Pandemic Quarantine

Pairwise comparisons (Table 3) suggest that there are significant differences between two measures of executive dysfunction (DEX). T1 (before the pandemic) not differ from T2 (during the pandemic) (*M*_*Diff*_ = 0.146, *p* = 1.000), but there are significant differences with T3 (after pandemic) (*M*_*Diff*_ = 4.313, *p* = 004). Participants scored a mean of 47.31 in December 2019 and 43 in July 2020. Significant differences were also observed between T2 (during the pandemic) and T3 (after the pandemic) (*M*_*Diff*_ = 4.167, *p* = 000), with T2's mean being 47.16 and T3's mean being 43. (Table 2 and Fig. 1a).

**Table 3:** Pairwise comparison of mean autism traits severity, executive dysfunction, balance, gait and well-being during COVID-19 Pandemic Quarantine

	*M* diff	*SE* diff	*p*	95% IC Diff	
				Lower	Upper
DEX					
T1 vs. T2	.146	1.267	1.000	− 2.999	3.291
T1 vs. T3	4.313	1.272	.004**	1.154	7.471
T2 vs. T3	4.167	.996	.000***	− 3.291	2.999
DIBAS-R					
T1 vs. T2	− 2.824	.839	.004**	− 4.902	− .745
T1 vs. T3	− .667	.721	1.000	− 2.454	1.121
T2 vs. T3	2.157	.847	.042*	.059	4.255
TINETTI-BALANCE					
T1 vs. T2	− .114	.291	1.000	− .839	.612
T1 vs. T3	1.136	.579	.169	− .307	2.580
T2 vs. T3	1.250	.463	.030*	.096	2.404
TINETTI-WALKING					
T1 vs. T2	− .023	.023	.969	− .079	.034
T1 vs. T3	− .273	.321	1.000	− 1.072	.527
T2 vs. T3	− .250	.320	.440	− 1.048	.548
PWI					
T1 vs. T2	2.077	1.307	.361	− 1.197	5.351
T1 vs. T3	− 1.641	1.289	.632	− 4.870	1.588
T2 vs. T3	− 3.718	1.148	.007**	− 6.592	− .844

*p < .05 **p < .01 ***p < .001. (Adjustment for various comparisons: Bonferroni)

*DEX* Dysexecutive Questionnaire, *DIBAS-R* Diagnostic Behavioral Assessment for Autism Spectrum Disorder- Revised, *TINETI-BALANCE and TINETI-WALKING* The Tinetti Assessment Tool, *PWI* Personal Wellbeing Index

Regarding the severity of autistic traits (DIBAS-R), pairwise comparison (Table 3) suggests that there are significant differences between the T1 (pre-pandemic) and T2 (during pandemic) measure (*M*_*Diff*_ = − 2.824, *p* = 004) and between T2 and T3 (post-pandemic) (*M*_*Diff*_ = 2.157, *p* = 042). Although no significant differences were observed between the T1 and T3 measure (*M*_*Diff*_ = − 0.667, *p* = 1.000), as symptomatology worsens at the 2nd measure and decreases again at the end (Table 2 and Fig. 1b).

The pairwise comparison (Table 3) of walking development measured through the Tinetti does not show significant differences between any of the measures (T1, T2 and T3). A positive trend in walking is perceived (Fig. 1c), although the results are not statistically significant.

Pairwise comparison (Table 3) of the balance measured through the Tinetti only shows significant differences between the T2 (during confinement) and T3 (after confinement) measurements (*M*_*Diff*_ = 1.250, *p* = 030). Therefore, it can be said that the equilibrium significantly worsens after confinement (Fig. 1d).

Finally, regarding emotional well-being, the pairwise comparison (Table 3) also shows significant differences between measure T2 (during confinement) and T3 (after confinement) (*M*_*Diff*_ = − 3.718, *p* = 007). In this case, a worsening (without significant differences) of emotional well-being is perceived between T1 (before confinement) and T2, and a significant increase in this emotional variable when confinement is terminated (T3) (Table 2 and Fig. 1e).

## Discussion

Previous research reveals a higher likelihood in the ASD and ID population to suffer from problems arising from confinement. The results of this study are reviewed below in relation to these risk factors.

The results demonstrated a significant effect of time on the severity of ASD traits, executive dysfunction, balance and well-being, although these differences do not support the initial hypothesis for all the variables. In the case of Executive Functions, an improvement has been observed at the 3rd measurement time (July 2021). These results need to be analyzed in depth in longitudinal studies that address a longer period of time, because with the data we have we can only see an improvement in aspects such as planning, inhibition, flexibility or working memory after the period of confinement, which could be due to the benefit of the return to activities outside the home, specific interventions, interaction with more people, etc.

Regarding the severity of autism symptomatology, we can observe how it worsens and increases during confinement and when this ends, it decreases again, remaining practically the same as before the pandemic and confinement. These results are consistent with previous studies that point to the negative effect of confinement on these individuals, due to the interruption of medical and psychoeducational services (Baweja et al., 2022; Eshraghi et al., 2020).

Balance also shows a negative evolution after confinement, the scores remain practically the same before and during confinement, but decrease significantly in the third measure, making evident the negative effects of confinement in this area so important in the prevention of falls and autonomy of any individual. Previous studies indicated specific difficulties in the motor area in people with ASD (Lim et al., 2021), which in the case of the population studied in this research worsen even more as an effect of the immobilism caused by COVID-19.

Finally, regarding the well-being scale, the results show a decrease during confinement and a recovery in the third measure, as the well-being of the population is higher than before the pandemic. These results are also consistent with the study by Davidson et al. (2021), who speak of 72% of adults with ASD having detected a deterioration in mental health during the pandemic.

### Limitations

The present study has some important limitations since there was no control group to compare the evolution of the study population with people without ASD or ID. It is also considered important for future research to expand the population with people who have remained at home and not in day centers, in order to better assess the different effects of these two environments. Finally, to better analyze the effects of the pandemic, it will be necessary to continue evaluating its effects on this population, since not only confinement has repercussions on the quality of life of these people, it is necessary to continue with the longitudinal study to be able to offer support and adapt interventions to the new needs of these people.

## Conclusion

People with ASD and ID are especially vulnerable to changes in the environment, so the pandemic and confinement have had very negative effects on them. In this longitudinal study we have analyzed the variables of interest before, during and after the period of confinement, observing a significant decrease in balance in the latter measure, along with a deterioration in well-being and ASD symptoms in the period of confinement and an improvement in executive functions after confinement.

These findings lead us to warn about the increased probability of the incidence of falls in this population, so we should focus on implementing interventions aimed at recovering and enhancing these abilities. On the other hand, the negative effects of confinement on the core symptoms of ASD and the sense of well-being are confirmed, so that in the future if similar situations occur, it would be appropriate to take this into account, adapt and try to minimize the effects on these people.
